# Impact of Bio-Based (Tannins) and Nano-Scale (CNC) Additives on Bonding Properties of Synthetic Adhesives (PVAc and MUF) Using Chestnut Wood from Young Coppice Stands

**DOI:** 10.3390/nano10050956

**Published:** 2020-05-18

**Authors:** Francesco Marini, Florian Zikeli, Piermaria Corona, Vittorio Vinciguerra, Maria Chiara Manetti, Luigi Portoghesi, Giuseppe Scarascia Mugnozza, Manuela Romagnoli

**Affiliations:** 1 Department of Innovation for Biological, Agrifood and Forest System (DIBAF), University of Tuscia, 01100 Viterbo, Italy; f.marini@unitus.it (F.M.); vincigue@unitus.it (V.V.); lporto@unitus.it (L.P.); 2 Centro Studi Alpino (CSALP) of the University of Tuscia, 38050 Pieve Tesino, Italy; florian.zikeli@tuwien.ac.at; 3 Institute of Chemical, Environmental and Bioscience Engineering, TU Wien, 1060 Vienna, Austria; 4 CREA (Council for Agricultural Research and Economics) Research Centre for Forestry and Wood, 52100 Arezzo, Italy; mariachiara.manetti@crea.gov.it

**Keywords:** cellulose nanocrystals (CNC), tannin, new value chain, bioeconomy, short supply chain

## Abstract

Sustainability and ecotoxicity issues call for innovations regarding eco-friendly adhesives in the production of biocomposite wood materials, and solutions involving nano-scale and bio-based compounds represent a valid and promising target. One possible approach is to increase the performance of adhesives such as polyvinyl acetate (PVAc) or melamine-urea-formaldehyde (MUF) by means of nanoparticles in order to obtain a material with better mechanical and environmental resistance. When applying cellulose-based nanoparticles or tannin, the concept of a circular economy is successfully implemented into the forest/wood value chain, and chances are created to develop new value chains using byproducts of forestry operations. In this study, assortments coming from young sweet chestnut (*Castanea sativa* Mill.) coppice stands were utilized for the preparation of single lap joint assemblies using different commercial adhesives (PVAc, MUF) and cellulose nanocrystals (CNC) and tannin as additives. The results showed that addition of CNC and tannin to PVAc glue increased tensile shear strength in lap joint tests presenting a promising base for future tests regarding the addition of CNC and tannin in MUF or PVAc adhesive formulations. Unfortunately, the tested bio-based additives did not reveal the same encouraging results when tested in the wet state.

## 1. Introduction

In the last years the necessity to substitute fossil resources with renewable ones has become one of the most important issues. Wood plays a major role from this point of view, but major challenges are associated to its more frequent use. Major topics are to diversify the use of less known species and to boost the use of species of a short supply chain for the production of wood-based composite materials according to bioeconomy principles [1]. 

The market for biocomposites is expected to grow in future thanks to a combination of factors like sustainability and renewability of wood resources, the inherent mechanical resistance of wood [2,3,4] as well as the incentive to exploit, as much as possible, short supply chain wood and low quality material [2,5]. However, the exploitation of the short supply chain material and its implementation into operative processes, should be evidence-based [6]. Among the species in Italy, which are cultivated in coppice stands and of interest for the short supply chain, sweet chestnut *(Castanea sativa* Mill.) represents one of the most important players [3,7]. Actually, chestnut logs from coppice stands are mainly used as beams for structural purposes, while smaller shoots find application as poles in agriculture [1,7]. Chestnut wood is characterized by high durability [8,9], attractive aesthetics and mechanical resistance, especially when considering the ratio between its specific weight and its mechanical performance [10,11]. Indeed, a change in its geographical distribution can be expected in future occupying zones with higher altitudes and latitudes due to climate change [4,7]. However, chestnut wood poses certain challenges regarding its quality due to the presence of ring shake defects [11,12] that can only be partially lowered by appropriate cultivation techniques [13]. Thus, traditional uses of chestnut wood should be reconsidered and new applications might be focused on the production of biocomposite materials where, through the utilization of wood adhesives, small logs could also be processed into a final product with satisfying mechanical properties [14,15].

The most common adhesives in the 20th century used in industry for the manufacturing of wood composite panels (particleboards, plywoods, oriented strand boards) include urea-formaldehyde, melamine-urea-formaldehyde (MUF), phenol formaldehyde resins (PF), polyurethane (PU) and polyvinyl acetate adhesives (PVAc) [16]. PVAc adhesives showed advantages like polymerization under normal pressure, their safety and their great compatibility with wood. On the other hand PVAc is mechanically unstable and shows low resistance to moisture [17], which is restricting its utilization to indoor conditions.

The social demand for eco-friendly adhesives requires increased research efforts in testing less toxic, renewable and bio-based components in future adhesive formulations. Thus, substitution of certain components in synthetic adhesives by non-toxic bio-based compounds presents a valid and reasonable approach for the development of eco-friendly adhesive formulations, resulting in numerous literature reports [18,19,20,21,22,23,24,25,26]. 

Cellulose as the most abundant biopolymer on our planet has been used in many applications, especially in the form of nanocellulose like cellulose nanocrystals (CNC). Recent research includes the development of multifunctional coatings for wood-based composites like novel waterborne PU coatings [27], CNC-coating of glass fibers for fiber-reinforced composites [28] or even the preparation of non-toxic electroactive hydrogels [29]. Thanks to several advantages (i.e., high strength and low weight), CNC also represent a promising component for the development of innovative reinforced wood adhesives [26,30].

Other relevant green adhesive formulations contain tannins and were designed in order to reduce formaldehyde emissions [3,31]. Among hydrolysable tannins, chestnut tannin has demonstrated importance for industry [32], and when applying it as adhesive component for chestnut wood panels, a circular economy concept applied in forestry could be fully developed. In order to speed-up the process of utilization of natural compounds like cellulose or tannin, mixing them with synthetic adhesives can be an interesting step towards the development of fully bio-based adhesive systems.

Among synthetic adhesives, PVAc presents strong advantages such as water-solubility, biodegradability and excellent chemical resistance as well as the fact that it is considered as non-toxic for human health [33]. The big disadvantage is the poor resistance in wet conditions and at high temperatures. For this reason, several attempts with encouraging results were carried out in order to increase the performance of PVAc through the addition of CNC and other compounds [33].

In this study with the aim for increasing strength and resistance of PVAc under outdoor conditions, the influence of tannin and cellulose nanocrystals (CNC) as additives in PVAc was investigated and compared with another important synthetic adhesive system (MUF) which is suitable for outdoor applications as well as for structural purposes. Mechanical tests were carried out on single lap joint panels made from young chestnut shoots from coppice stands, in order to show the possibility of a sustainable use of wood from a short supply chain with eco-friendly adhesives. Further, as a new diagnostic method in wood technology, ultraviolet fluorescence (UVF) analysis as a non-destructive technique that takes advantage of the visible light response of surfaces illuminated by UV radiation was applied [34]. Non-invasiveness, practicality and immediate responses, thanks to newly developed LED light sources, make UVF one of the most used techniques in cultural heritage conservation sciences, but up-to-date no publications regarding wood analysis can be found. 

## 2. Materials and Methods 

### 2.1. Wood Material

Chestnut *(Castanea sativa* Mill.) wood derived from young chestnut trees with an age of 12–15 years are cultivated in coppice stands on Amiata Mountain, Tuscany, central Italy. Trunks with a length of 1.5 m were cut and from each trunk, sample logs of 40 cm length were taken in order to cut boards in radial direction with 2 cm thickness and 8 cm width. The boards were then cut in fiber direction and sanded to a final thickness of 5 mm, followed by conditioning in standard climate (temperature: 20 ± 2 °C, relative humidity: 65 ± 5%) to reach an equilibrium moisture content of about 12% according to [35].

### 2.2. Adhesives, Additives and Gluing Conditions 

Commercial PVAc adhesive for non-structural applications as well as a MUF adhesive system were used as reference formulations and as the base for the preparation of different formulations containing CNC, tannin, or both (Table 1). PVAc adhesive is classified as D3 according to [34], which means it is suitable for interior areas, with frequent short-term exposure to running or condensed water and heavy exposure to high humidity. The used MUF adhesive system is classified as class 1 according to EN 301:2013, which means it is suitable to be used for structural purposes with full exposure to weather.

### 2.3. Scanning Electron Microscopy (SEM) Analysis

CNC (chemically: cellulose hydrogen sulfate sodium salt) were purchased from CelluForce Inc. (Montreal, QC, Canada) and chestnut tannin (Saviotan) was used from Saviotan (Gruppo Saviola - Radicofani, Italy). The spray-dried CNC powder has a bulk density of 0.7 g/m^3^, a moisture content of 4–6% and a particle size spanning from 1 to 50 µm. CNC crystallites (particle diameter: 2.3–4.5 nm, particle length: 44–108 nm) have a hydrodynamic diameter of 70 nm. 

CNC and tannins, in powder form, were manually mixed into the PVAc and MUF adhesives. Solid contents of the additives, the modified formulations as well as the commercial adhesives are reported in Table 1. 

Bonding procedures were carried out according to instructions given by the adhesives’ manufacturers. After manual application of the requested amount of glue (130 g/m^2^ PVA and 250 g/m^2^ MUF) using an aluminum spatula, the chestnut wood tangential panels were bonded in parallel orientation and pressed for 90 min at 50 bar (5 MPa) at ambient temperature. After pressing, the glued samples were dried in standard climate at 20 °C and 65% relative humidity (RH) according to [35]. A set of 18 lap joint test pieces was prepared for each glue formulation given in Table 1.

After cutting to the requested dimensions for the sample holders, the lap joint samples were prepared at the Electron Microscopy Section (CIME) of the Large Equipment Center (CGA) of the University of Tuscia for morphological analysis using SEM. Samples were attached to aluminum stubs using conductive double-sided carbon tape and coated with gold using a Balzers MED 010 sputtering unit (Oerlikon Balzers, Balzers, Liechtenstein). SEM analysis was done using a JEOL JSM 6010 LA instrument (JEOL Limited, Tokyo, Japan), both in transversal and tangential sample direction, in order to investigate the respective glue-lines. Dimensions of glue-lines were determined on the SEM micrographs using Adobe Photoshop CS2 software package (Adobe Systems, San Jose, CA, USA). 

### 2.4. Mechanical Tests

**Figure 1:**
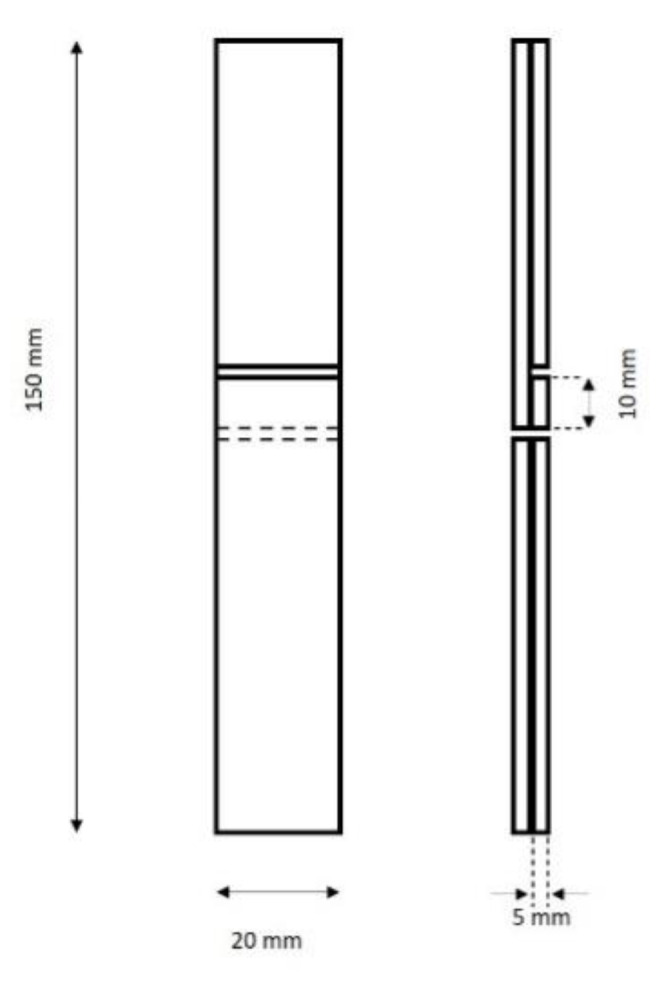
Lap joint sample for the mechanical shear test.

The glued and conditioned wood panels were cut to the final dimensions of the lap joint test pieces (150 × 20 × 10 mm) in order to prepare 8 test pieces for each of the treatments A1, A2 and A3 (Table 2), which were subjected to tensile shear strength ($f_{V}$) tests in a universal testing machine (Zwick Roell Z050, Germany) following [35] (Figure 1). 

Tensile shear strength ($f_{V}$) of the samples was calculated using Equation (1), where $F_{max}$ is the total force at failure in Newton (N) and A is the glued test surface (200 mm^2^).

(1) $$f_{V}=F_{max}/A$$

According to [35], an estimation of the test surface covered by wood fibers (graded as 0%, 10%, 20%, etc., to 100% wood failure) was carried out for each sample. For each treatment and glue formulation, average wood failure with standard deviation was determined. Data were analyzed using Past 3 software. Since the data were not normally distributed, mechanical differences between the different gluing applications were explored through a post-hoc nonparametric test (Mann–Whitney U test) of the variables: shear strength values and wood failure percentage. The values above 95% of statistical probability were taken into account.

### 2.5. Ultraviolet Fluorescence Photography 

UVF instrumentation consisted of a UV-A LED light source (Madatec srl, Pessano con Bernago, MI, Italy) with a λ_max_ of 365–370 nm, a mirrorless digital photo camera with 18 Mpx resolution (Madatec) and a HOYA UV&IR Cut filter (Kenko Tokina Co., Ltd., Nakano, Japan). For an excellent result, it is important to apply the light source with an angle of 30–45° in respect to the samples for homogeneous illumination and to ensure that the visible component is the only one emitted from the examined surface. Further, working in total darkness is mandatory and photos are taken with long exposure times in order to record fluorescence deriving from the investigated lignocellulosic material with multiplied intensity.

## 3. Results and Discussions

### 3.1. Morphological Characterization Using Scanning Electron Microscopy

SEM analysis allowed for the determination of an average glue-line thickness over the length of the analyzed sample, representative values measured on one sample are reported in Table 3. All glue-lines of the different glue formulations had an average thickness around 80–100 µm. In samples C (PVAc-CNC-T) and D (MUF) the respective glue-lines were thicker than observed for the rest of the samples. In the case of sample D this could be due to the higher amount of adhesive applied. Interestingly, the formulations with added CNC (samples B and E) showed a thinner bond line compared to their respective control formulations (samples A and F). This could indicate a structurizing effect of CNC bundles, providing a kind of scaffold for the glue that limits its expansion during polymerization. In the case of the MUF adhesive system, CNC methylol side-chains potentially became involved in MUF polymerization mechanisms creating methylene bridges. In the case of PVAc, interactions between the glue and CNC are supposed to happen via hydrogen bonding between PVAc acetyl and CNC OH groups. 

**Figure 2:**
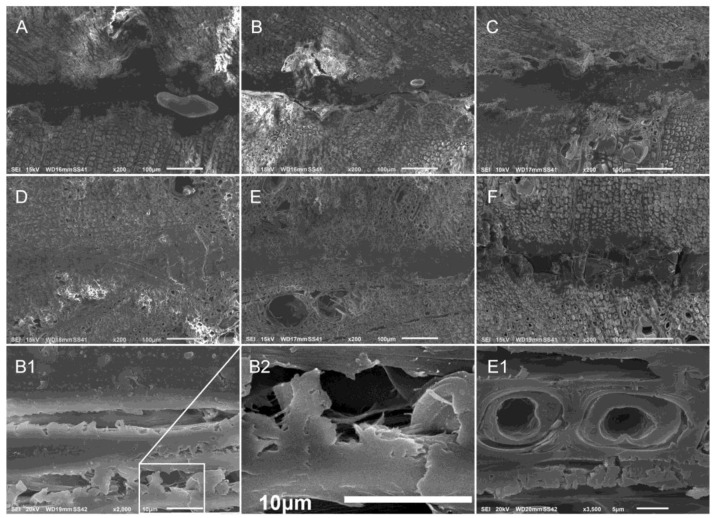
SEM images of transversal cuts of the glued samples using the different adhesive formulations: PVAc (**A**), PVAc-CNC (**B**), PVAc-CNC-T (**C**), MUF (**D**), MUF-CNC (**E**), T-CNC (**F**), (**B1)** tangential cut of sample B, (**B2**) magnification of the boxed area of B1 and (**E1**) magnification of a tangential cut of sample E.

Inclusions, which were classified as air bubbles, were observed inside the glue-lines of the samples A and B (Figure 1). In sample A (PVAc), the inclusions were rather large with a length of 175 µm, while the one in the glue-line in sample B (PVAc-CNC) was minor (40 µm). Both cases were apparently caused by surface irregularities of the wood panels due to collapsed vessels in the ring porous structure of chestnut wood, which were observed near to the inclusions (Figure 2).

In sample A (PVAc) the glue-line was widely attached to the wood substrate. When CNC (sample B) and CNC plus tannin (sample C) were added to PVAc, the respective glue-lines were in part detached from the interphase with the wood surfaces. Adding CNC reportedly increased the viscosity of PVAc [14,31,35] resulting in less penetration of the glue into the wood surface, thus increasing the risk for delamination.

Sample F was glued with an adhesive formulation consisting solely of tannin, CNC and water. The respective glue-line was very well attached to the wood panels’ surfaces. However, creeps were detected inside the glue-line, indicating unsuccessful curing of this adhesive formulation (Figure 2). Furthermore a possible auto-condensation in tannins could have affected the cohesion in the bond layer, negatively affecting the wood bonding process [36]. During mixing of the formulation T-CNC, a dramatic viscosity increase was observed preventing successful homogenous distribution over the complete wood panel surface and thus limiting gluing performance, as it was also observed in other investigations [37].

**Figure 3:**
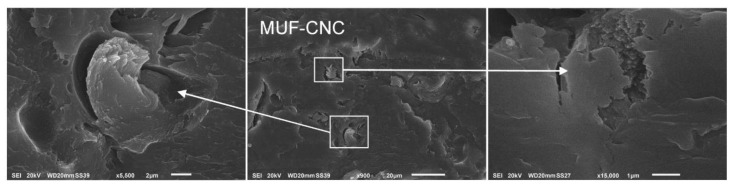
Bundles of cellulose nanocrystals (CNC) in a higher magnification of the glue-line of sample E (MUF-CNC).

In contrast to samples B, C and F, the glue-lines of sample D (MUF) and E (MUF-CNC) were considered as nearly perfect, as they presented well developed interphases with the wood panels’ surfaces and had no air inclusions inside the glue-line, confirming reports regarding the high compatibility of MUF resins with wood and their strong adhesion to cellulose fibers due to a lower viscosity of MUF adhesives in general [14]. In sample E successful incorporation of the added CNC inside the MUF adhesive matrix was observed, firstly, maintaining its viscosity during mixing as well as regarding a successful glue penetration inside the wood cells. In fact, in sample E the adhesive penetrated very deeply following the parenchymatic rays, which facilitated the spreading of the adhesive (Figure 3, E1). Furthermore, due to the higher dispersion of MUF and MUF-CNC in the wood matrix, the limits of the interphase layer adhesive/wood are less identifiable. 

At higher magnifications, CNC in the form of bundles was detected widespread in the adhesive matrix both in PVAc and MUF, similarly to the results obtained by [38]. Inside the glue-line of sample B (PVAc-CNC), at higher magnifications, fibrous structures can be observed and in sample E (MUF-CNC) strong bundles of CNC fibers are visible (Figure 2B1,B2,E1, and Figure 3).

**Figure 4:**
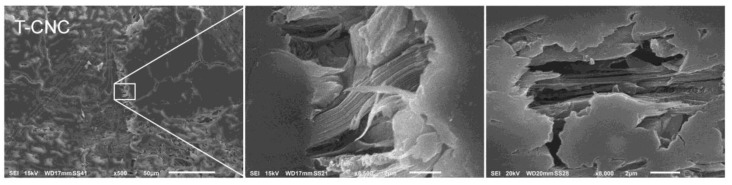
Creeps inside the glue-line of sample F (T-CNC) showing bundles of cellulose nanocrystals (center and right).

Indeed, in the SEM images of adhesive formulation F (T-CNC), CNC bundles were detected too, apparently in parallel orientation to the wood panel fibers which could provide additional strength to the glue-line and compensate for the observed creeps (Figure 4, center and right).

### 3.2. Mechanical Properties of Laminated Veneer Lumber Samples

Table 4 shows the average shear strength of the lap joint test specimens prepared using six different adhesive formulations, after the respective treatments, A1, A2 and A3 (Table 2). The obtained values were also compared with their respective minimum and maximum shear strength results, their variability and the percentage of wood failure is reported in Table 4. Statistical significance of the values is reported in Table 5 (only values over 95% are showed). The results will be discussed in the following sections according to the respective treatments.

### 3.3. Dry Conditions

Mechanical strength of PVAc is under the required limit for a D3 glue of 10 MPa, the difference could be attributed to a sub-optimal spread of the adhesive on the surface, which was carried out by hand for the scientific purpose of the research. 

In the formulation PVAc-CNC, relative to its control (PVAc), the added CNC seems to positively affect the mean value of the shear strength after treatment A1. This should be in agreement with the results of [35], who supposed an interlocking effect due to increased cross-linking of the methylene groups from PVAc with hydroxyl groups from CNC as well as cellulose from wood. However, the shear strength increase is not statistically significant (Table 5), indeed, there is remarkable default in some samples regarding the adhesion in the interphase layer.

Weak interphases were also visible in several samples when UVF analysis was applied. UV microscopy was shown to be effective in detecting adhesive distribution in the work of other authors [39]. However, this is the first time to our knowledge that UV radiation is applied on macro-scale samples using the presented protocol. 

**Figure 5:**
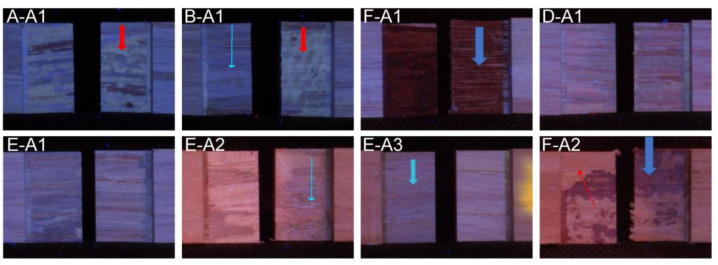
PVAc after treatment A1 (**A-A1**): arrow indicates PVAc presence; PVAc-CNC (**B-A1**) after treatment A1: red arrow indicates glue presence, thin blue arrow blue indicates spots where CNC are supposed to be grouped; T-CNC after treatment A1 (**F-A1**): blue arrow for dark color of tannin; MUF after treatment A1 (**D-A1**); MUF-CNC (**E-A1**) after treatment A1: a more uniform color is visible; MUF-CNC after treatment A2 (**E-A2**): the arrow indicates bluish spots related to CNC; MUF-CNC after treatment A3 (**E-A3**): CNC blue spots are homogenously dispersed; T-CNC after treatment A2 (**F-A2**): blue arrow indicates residues of tannin, thin arrow indicates wood.

In this study, accordingly, the UV photographs (Figure 5A-A1) revealed presence of PVAc glue on both sides of the lap joints and wood failure was indicated by brown spots. When CNC is added, the partial delamination of some samples gets evident because adhesive is visible only on one side of the lap joint (Figure 5B-A1). Furthermore, in Figure 5B-A1 small particles of blue-whitish color seem apparent (arrow), which could be related to a strong agglomeration of CNC which was also reported elsewhere [40]. 

The adhesive formulation PVAc–CNC-T does not improve adhesion, because the determined shear strength in the dry state A1 was not significantly higher compared to pure PVAc. Shear strength results in this formulation showed rather high variability. However, when excluding the values below a threshold of 2 N/mm^2^, minimum shear strength values were evidently increased reaching 9.25 N/mm^2^, indicating a big potential. In conclusion, CNC and CNC-tannin as additives could increase the performance of PVAc adhesive, but glue handling during application onto the wood panels was identified as a crucial point. Further positive evidence was the percentage of wood failure, which, when the spread of the glue is optimal, is higher compared to its control, considering both minimum and maximum values (Table 4). 

The highest average lap joint tensile shear strength was observed for the adhesive formulations MUF and MUF-CNC, reaching almost 12 N/mm^2^. Compared to its control, the CNC-enriched formulation demonstrated very similar values after all three treatments, as assessed by the descriptive statistic and statistical analysis.

Considering the polymerization mechanism of MUF resins, where methylol groups and primary amines from urea and melamine react in a condensation reaction [41], the high presence of side-chain methylol groups in cellulose was expected to promote cross-linking of CNC with amine groups of urea and melamine during the gluing process, leading to profound integration of CNC into the structure of the MUF resin. Since for methylolation of urea and melamine the use of formaldehyde is essential, CNC could present a promising substitute for formaldehyde moieties in MUF resin formulations, offering their already present methylol side-chains for the addition of urea or melamine via condensation reactions creating methylene or methylene-ether bridges. The comparable performance of wood panels glued with MUF and MUF-CNC, respectively, was confirmed by their similar wood failure results. Furthermore, UVF photographs revealed a homogeneous distribution of both adhesive formulations MUF and MUF-CNC on both contact sides of the lap joint (Figure 5D-A1,E-A1). When using the glue formulations MUF and MUF-CNC, shear strength values showed the lowest standard deviations, demonstrating highly homogeneous glue-lines along the length of the glued panels. Although CNC-addition to a MUF adhesive system did not result in higher shear strength under dry conditions, substitution of a part of the applied synthetic adhesive system by a renewable compound must be highlighted when aiming for more eco-friendly adhesive systems.

### 3.4. Wetting (A2) and Wetting-Reconditioning (A3)

In the wet-state (treatment A2), shear strength was substantially reduced for all tested formulations.

After treatment A2, the addition of CNC to PVAc resulted in a slight shear strength increase, but in contrast, after wetting and re-conditioning (A3) much lower shear strength was observed. After A3 treatment, it becomes evident that PVAc hardens as a result of coagulation of dispersed adhesive particles after water is released into the surrounding material, giving a final shear strength which is comparable to the initial one [17]. The addition of CNC seems to introduce a disadvantage for this process because of a remarkable decrease in shear strength after treatment A3. From this point of view, it is supposed that the thermoplastic behavior of the glue facilitates a cross-linking with CNC making the adhesive less available to cling to the wood tissue. Even if the result is not positive, other benefits of CNC-reinforced PVAc glues such as superior heat resistance have been reported in literature by other authors [42], which could be considered an advantage. Interestingly, when a variant is introduced like using additional tannin reinforcement (PVAc-CNC-T), a slightly better performance was observed. In fact, compared to the addition of only CNC to PVAc, tannins lead to an increase of shear strength after treatment A3 as well as a moderate increase of wood failure after both treatments A2 and A3. The effect of tannin as an efficient glue additive has already been proved elsewhere [30,43] and it is confirmed at least in part in our investigation. In conclusion, it can be affirmed that the modified formulations PVAc-CNC and PVAc-CNC-T could hardly compete with the commercial adhesives in wetting and wetting-reconditioning processes.

MUF adhesives performed differently and after adding CNC to the MUF adhesive system, no evident effect on the measured shear strength in the wet-state was observed. Nevertheless, a higher percentage of wood failure was noted which could indicate better performance in outdoor conditions, supported by a low variance of the different samples glued with this formulation (Table 4, CV). Analysis of the UVF photographs further showed rather homogenous distribution of CNC (blue absorption) inside the glue-line (Figure 5E-A3) and this result could be related to the good dispersion of cellulose nanofibrils in UF resins observed in several cases as reported in [44].

Samples glued with the adhesive formulation which contained solely tannin and CNC (T-CNC) demonstrated lower shear strength after treatment A1 and samples were completely delaminated after treatments A2 and A3, supposedly due to tannin solubility in water. For this reason, tensile shear strength measurements were impossible. The strong coloring of tannin is visible in dark color, while in other parts (arrow) raw wood can be observed, testifying that the glue was partly washed out during wetting treatment A2 (Figure 5F-A1,F-A2).

## 4. Conclusions

Many wood resources can be exploited more efficiently than they actually are, like small logs of chestnut wood that should be considered as a promising resource for a growing market of biocomposite materials. Lignocellulosic resources therefore can become a pillar in developing new value chains following basic concepts of a bio-circular economy. High abundance of cellulose in nature and the need for more eco-friendly adhesives qualify CNC as a reasonable additive. In our research work, CNC-addition to the synthetic adhesive PVAc resulted in increased mechanical performance as assessed by lap joint tensile shear strength tests under dry conditions. Addition of both CNC and tannin together to PVAc glue also increased shear strength of the prepared lap joint test specimen compared to the use of pure PVAc glue. In wet and in reconditioned samples, tannin successfully mitigated the negative effect of solely CNC-addition to PVAc. Furthermore, CNC proved to be very compatible with MUF resin, and future works have to be carried out to test its potential role on the further reduction of formaldehyde emissions.

## Figures and Tables

**Table 1:** Adhesive formulations based on commercial adhesives and cellulose nanocrystals and tannins as additives used for gluing experiments.

Adhesive Formulations	Adhesive ID	Sample Label	CNC (%)	Tannin (%)	Solid Content (%)	Viscosity (mPas)
Polyvinyl acetate adhesive (PVAc)	PVAc	A	0	0	52–54	13,000
PVAc + Cellulose Nanocrystals (CNC)	PVAc-CNC	B	5	0	54–56	n.d.
PVAc + CNC + Tannin (T)	PVAc-CNC-T	C	5	5	60–65	n.d.
Melamine-urea-formaldehyde adhesive (MUF) (ready to use)	MUF	D	0	0	60–62	10,000–25,000
MUF + CNC	MUF-CNC	E	5	0	63–65	n.d.
T + CNC + water	T-CNC	F	5	70	68	n.d.

**Table 2:** Type and duration of treatment prior to tensile shear testing (adapted from [35]).

Designation	Treatment
**A1**	No treatment other than conditioning in standard climate (20/65)
**A2**	4 days soaking in cold water at (20 ± 5) °C Samples tested in the wet state
**A3**	4 days soaking in cold water at (20 ± 5) °C Reconditioning in standard climate (20/65) to original mass Samples tested in the dry state

**Table 3:** Glue-line thickness of the tested adhesive formulations in representative parts of the samples.

Sample Label	Adhesive ID	Glue-Line Thickness (µm)
**A**	PVA	92.4
**B**	PVA-CNC	64.1
**C**	PVA-CNC-T	101.8
**D**	MUF	140.0
**E**	MUF-CNC	82.8
**F**	T-CNC	92.4

**Table 4:** Mean shear strength with standard deviation (SD), coefficient of variation (CV), minimum and maximum shear strength, number (#) of samples with a shear strength under 2 N/mm^2^, invalid shear strength test samples, mean wood failure (WF) percentage with standard deviation, minimum and maximum wood failure.

Treatment Type	Adhesive ID	# Samples	Mean Shear Strength ± SD (N/mm^2^)	CV (%)	Shear Strength Min	Shear Strength Max	# Samples under Threshold 2 N/mm^2^	Invalid Test Samples	Mean WF ± SD (%)	WF Min (%)	WF Max (%)
**A1**	PVAc	8	5.32 ± 2.06	38.72	2	7.82	0	0	50 ± 25.0	10	60
	PVAc-CNC	5	7.54 ± 3.02	40.05	2.82	11.24	0	0	40 ± 19.2	20	80
	PVAc-CNC-T	5	6.22 ± 4.52	72.66	9.25	9.99	2	0	40 ± 30.3	0	80
	MUF-CNC	8	11.95 ± 1.67	13.97	9.83	13.73	0	0	90 ± 9.1	70	100
	MUF	8	12.19 ± 1.71	14.02	9.96	14.34	0	0	90 ± 7.5	80	100
	T-CNC	6	5.04 ± 2.22	44.04	4.84	5.24	1	3	8.3 ± 7.5	0	20
**A2**	PVAc	4	0.85 ± 0.54	63.52	0.38	1.61	0	0	20 ± 5.7	20	30
	PVAc-CNC	5	0.92 ± 0.8	86.95	0.23	1.79	0	2	10 ± 5.4	10	20
	PVAc-CNC-T	5	0.56 ± 0.34	60.71	0.17	0.95	0	1	30 ± 8.9	20	40
	MUF-CNC	5	4.31 ± 3.46	80.27	0.83	9.01	2	0	80 ± 5.4	80	90
	MUF	5	4.62 ± 1.17	25.32	3	6.01	1	0	70 ± 8.9	60	80
	T-CNC	3	INVALID	-	-	-	-	3	-	-	-
**A3**	PVAc	2	5.33 ± 3.53	66.22	2.82	7.82	0	0	70 ± 21.2	60	90
	PVAc-CNC	6	2.58 ± 3.33	129.0	0.15	8.98	2	0	30 ± 16.4	20	60
	PVAc-CNC-T	5	4.39 ± 2.35	53.53	1.23	6.74	1	0	60 ± 28.2	20	80
	MUF-CNC	5	10.51 ± 2.45	23.31	8.25	12.95	0	0	90 ± 17.8	60	100
	MUF	4	10.8 ± 2.77	25.64	7.56	13.94	1	0	80 ± 12.2	60	90
	T-CNC	6	INVALID	-	-	-	-	6	-	-	-

**Table 5:** Mann–Whitney test on shear strength value. *** *p* < 0.001, ** *p* < 0.01, * *p* < 0.05.

PVAc-CNC	PVAc	PVAc-CNC-T	MUF	MUF-CNC	T-CNC
**PVAc-CNC**			A2 *	A3 *	A1 *
	**PVAc**		A2 * A1 ***	A1 ***	A1 *
		**PVAc-CNC-T**	A2 * A1 **	A3 * A2 * A1 **	A1 * A2 *
			**MUF**		A1 ** A2 *
				**MUF-CNC**	A1 **
					**T-CNC**
